# Concise Review: Bone Marrow-Derived Stem/Progenitor Cells in Cutaneous Repair and Regeneration

**DOI:** 10.1002/stem.420

**Published:** 2010-05

**Authors:** Yaojiong Wu, Robert C H Zhao, Edward E Tredget

**Affiliations:** a Life Science Division, Graduate School at Shenzhen, Tsinghua UniversityBeijing, People's Republic of China; b Department of Cell Biology, Center of Excellence in Tissue Engineering, Institute of Basic Medical Sciences, Chinese Academy of Medical Sciences and School of Basic Medicine, Peking Union Medical CollegeBeijing, China; c Wound Healing Research Group, Department of Surgery, University of AlbertaEdmonton, Alberta, Canada

**Keywords:** Cutaneous regeneration, Wound healing, Mesenchymal stem cells, Fibrocytes, Endothelial progenitor cells

## Abstract

Our understanding of the role of bone marrow (BM)-derived cells in cutaneous homeostasis and wound healing had long been limited to the contribution of inflammatory cells. Recent studies, however, suggest that the BM contributes a significant proportion of noninflammatory cells to the skin, which are present primarily in the dermis in fibroblast-like morphology and in the epidermis in a keratinocyte phenotype; and the number of these BM-derived cells increases markedly after wounding. More recently, several studies indicate that mesenchymal stem cells derived from the BM could significantly impact wound healing in diabetic and nondiabetic animals, through cell differentiation and the release of paracrine factors, implying a profound therapeutic potential. This review discusses the most recent understanding of the contribution of BM-derived noninflammatory cells to cutaneous homeostasis and wound healing.

## INTRODUCTION

Recent studies indicate that cells from the bone marrow (BM) contribute a significant proportion of cells in the skin [1,2]. The normal skin has long been known to contain BM-derived cells that are involved in host defense and inflammatory processes, including wound healing. However, recent studies strongly suggest that the BM contributes not only inflammatory cells, but also keratinocytes and fibroblast-shaped cells to the skin [1–3]. Similar to leukocytes in trafficking, stem/progenitor cells derived from the BM could home to injured tissues and participate in the repair/regeneration [4,5]. Moreover, culture expanded bone marrow-derived mesenchymal stem cells (BM-MSCs) have been shown to promote the healing of diabetic wounds [6,7], implying a profound therapeutic potential for skin defects such as chronic wounds and burns.

There are two main branches of stem cells in the BM, including hematopoietic stem cells (HSCs) and mesenchymal stem cells (MSCs). Adult BM-derived HSCs have long been recognized as a precursor to all blood cell lineages including erythrocytes, platelets, and white blood cells. Additionally, HSCs may also give rise to fibrocytes [8,9] and endothelial progenitor cells (EPCs) [10,11]. A common feature of hematopoietic lineage cells is the expression of the cell-surface antigen CD45, with the exception of mature red blood cells and their immediate progenitors. Although some studies suggest that HSCs may also give rise to nonblood cells such as hepatocytes [12], smooth muscle cells, and cardiac myocytes [13], subsequent studies suggest that these phenomena may only represent occasional events [14,15].

BM-MSCs, which are also referred to as BM mesenchymal stromal cells or marrow stromal cells, are self-renewing and expandable stem cells [16–18]. Although present as a rare cell population in the BM, representing about 0.001% to 0.01% of the nucleated cells, about 10-fold less abundant than HSCs, MSCs are expandable in culture and multipotent, capable of differentiating into several cell types [17,18]. To compare and contrast study outcomes from different research groups, the Mesenchymal and Tissue Stem Cell Committee of the International Society for Cellular Therapy proposed a minimal criterion to define human MSCs. First, MSCs must be plastic-adherent when maintained in standard culture conditions. Second, MSCs must be lineage-negative and express CD105, CD73, and CD90. Third, MSCs must differentiate to at least osteoblasts, adipocytes, and chondroblasts in vitro [19]. Numerous animal studies suggest that BM-MSCs contribute to the repair/regeneration of a variety of injured tissues including the myocardium [20], cardiac valves [21], bone [22], tendon [23,24], cartilage [25], and meniscus [26].

## BM-DERIVED FIBROBLAST-SHAPED CELLS IN THE DERMIS

Several recent studies suggest that cells from the BM contribute to a significant proportion of the cells in the skin [1,2,27]. In one study, using a chimeric mouse model in which the BM of C57BL mice was reconstituted with BM stem cells from transgenic mice expressing enhanced green fluorescent protein (EGFP), EGFP^+^ cells derived from the BM were found in the normal skin and actively participated in wound healing. Impressively, as many as 15%–20% of the spindle-shaped dermal fibroblast-like cells in the noninjured normal skin were EGFP^+^, suggestive of BM origin, and over two thirds of them were negative for CD45 [1], implying of nonhematopoietic cells. Consistent with the above observation, in another study, using a similar mouse model, green fluorescent protein^+^ (GFP^+^) BM-derived cells accounted for 8.7% of total fibroblast-shaped cells in the normal skin, and the proportion of GFP^+^ fibroblast-shaped cells markedly increased in fibrotic lesions caused by cancer cell implantation (59.7% ± 16.3%) or wounding (32.2% ± 4.8%). Immunofluorescence analysis showed that these GFP^+^ spindle-shaped BM-derived cells expressed collagen type I, and only about 50% of them were also positive for CD45 [27]. Taken together, these studies suggest that about half of the BM-derived fibroblast-shaped cells in the skin were of nonhematopoietic lineages, suggestive of noninflammatory cells. This finding has markedly improved our understanding to the contribution of BM cells in cutaneous physiology and pathology, and opened a new avenue to study the mechanisms of cutaneous homeostasis and wound healing.

## IDENTITIES OF BM-DERIVED FIBROBLAST-LIKE CELLS IN THE DERMIS

The identities of BM-derived fibroblast-like cells in normal skin and in the healing wound remain largely to be determined. Theoretically, several types of cells derived from the BM may exhibit spindle-shaped fibroblast-like morphology, including fibroblasts, MSCs, macrophages, fibrocytes, and EPCs. Macrophages, fibrocytes, and EPCs are currently considered to originate from HSCs and hence express CD45. However, it should be noted that the expression of CD45 in fibrocytes and EPCs decrease on differentiation or maturation of the cells, and this makes it difficult to recognize them by this marker. It is unclear whether fibroblasts with similar properties to those in the connective tissue exist in the BM as an independent cell population. Thirty years ago, a population of fibroblast-like cells were found in the BM, which were adherent to plastic tissue culture dishes and formed colonies in an alpha medium supplemented with serum. The cells were negative for coagulation factor VIII but expressed collagen types I and III and fibronectin, which were also expressed by dermal fibroblasts. The cells were thus termed BM fibroblasts [28]. In late last decade, cells derived from the BM with similar isolation and culture methods as used in the above study were found to be able to differentiate to osteoblasts, chondrocytes, and adipocytes, and were thus named MSCs [17,18]. Theoretically, MSCs are stem cells with multiple differentiation possibilities whereas fibroblasts are specialized cells and unable to differentiate into other cell lineages, but both of them are similar in cell morphology and bear a similar group of surface markers such as CD90, CD44, CD105, and CD73 [29], which are markers considered typical for MSCs. In our experience, when murine BM-derived lineage negative adherent cells are culture expanded in a medium (α-minimal essential medium, α-MEM) supportive to the growth of both MSCs and dermal fibroblasts, over 95% of such cultured cells are positive for Sca-1. Sca-1 is a marker expressed in murine MSCs derived from BM and several other tissues, but is not expressed in dermal fibroblasts [29], and probably other tissue fibroblasts as well. Moreover, BM-MSCs and dermal fibroblasts are distinctively different in expression of a variety of cytokines and in wound healing (see more discussion in the subsequent sections). These data suggest that the vast majority of fibroblast-like cells in the BM are MSCs, and conventional fibroblasts are likely not to exist in the BM as an independent cell population.

As MSCs and fibroblasts are similar in morphology and express a similar group of surface antigens (Sca-1 is not expressed in humans), it is difficult to distinguish them in tissues, especially in humans. Collagen type I expression was considered to be a typical feature of dermal fibroblasts by some investigators [27]. But other studies have indicated that it is also expressed in fibrocytes [8,9], macrophages [30], and MSCs [1]. One study suggests that BM-MSCs express collagen type III, whereas dermal fibroblasts do not [1]. Collagen type I is the predominant form of collagen in normal human skin, and the expression of collagen type III increases markedly early in normal wound healing. Therefore, collagen type III is considered to be particularly important for wound healing. Interestingly, a study showed that collagen type III was produced by cells derived from the BM, but not fibroblasts residing in the dermis [1], implying an indispensable role for BM-derived cells in wound healing. Unlike MSCs and fibroblasts, BM-derived macrophages and fibrocytes can be distinguished from dermal fibroblasts and BM-MSCs by their expression of CD45, and EPCs can be recognized by their expression of endothelial lineage markers such as VEGFR2 and CD31 [10,11] (Table 1). Despite their morphological similarities, dermal fibroblasts, MSCs, macrophages, fibrocytes, and EPCs have exhibited distinctive biological and physiological activities (Table 1).

**Table 1:** Properties and activities of dermal fibroblast-like cells

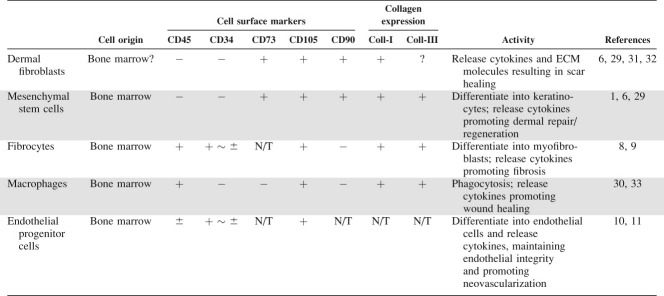

Abbreviations: −, negative; +, positive; ±, expression decreases or disappear on cell maturation; ?, controversial; (+), + ∼ ±, Positive to weak positive; ECM, extracellular matrix; N/T, not tested; Coll-I, collagen type I.

## MSCS FROM THE SKIN AND OTHER TISSUES RESEMBLE BM-MSCS

“Mesenchyme” designates the developing loose connective tissue of an embryo, mainly derived from the mesoderm, and giving rise to a large part of the cells of the connective tissue in the adult [34]. Many mesenchymal tissues contain committed lineage-directed mesenchymal precursor cells, which participate in local regeneration, such as the satellite cell in skeletal muscle or the adipocyte progenitors of adipose tissue. Uncommitted mesenchymal progenitors, capable of giving rise to other types of mesenchymal tissue other than the one they are present in, have also been found in scalp tissue [35], skeletal muscles [36], bone [37], and adipose tissue [38,39]. Cultured scalp MSCs expressed a similar group of surface antigens as BM-MSCs. Moreover, scalp MSCs exhibited similar differentiation potential along osteogenic, chondrogenic, and adipogenic lineages, compared with human BM-MSCs [35]. Analysis of MSCs derived from other non-BM sources has also revealed a strong intergroup correlation of MSCs as they all express antigens, which have been considered typical markers of BM-MSCs such as CD90, CD105, and CD73, despite an inconsistent nomenclature and lack of standardized protocols for cell isolation and culture [38–42].

## CIRCULATING MSCS

One major question arising from the similarity of BM-MSCs to MSCs isolated from other tissues is whether BM constitutes a source of MSCs capable of participating in the repair and regeneration of multiple tissues of mesenchymal origin in the adult organism. Systemic infusion of BM-MSCs in baboons after lethal total body irradiation results in long-term engraftment of the cells in multiple organs including the skin, intestines, liver, lungs, thymus, and kidneys [4], indicating that BM-MSCs have the ability to traffic and survive in blood and migrate to injured tissue. Many investigators have attempted to investigate circulating MSCs. Several recent studies suggest that there is a subpopulation of cells in the peripheral blood of mice and humans, which resemble BM-MSCs. Cells that exhibit similar surface antigen profiles and differentiation potency to BM-MSCs have been consistently isolated from umbilical cord blood [41,43,44], indicating the existence of circulating MSCs in the fetus. In adults, a putative circulating MSC population (defined as CD45^–^/CD34^–^ nucleated cells) has been found in the peripheral blood of mice [45] and humans [46] by fluorescence-activated cell sorting analysis, which accounted for 2–8% of total nucleated cells in the peripheral blood [45,46]. However, isolation and cultivation of this cell population from the peripheral blood appears to be a difficult task and is frequently unsuccessful. MSC-like cells in the peripheral blood were first characterized in 2001 using isolation and culture methods similar to those used for BM-MSC in four species of adult animals (mouse, rabbit, guinea pig, and human) [47]. Circulating MSCs were also found in the peripheral blood of patients receiving granulocyte colony-stimulating factor (G-CSF) or granulocyte-macrophage colony-stimulating factor (GM-CSF) treatment [48]. However, other research groups have not consistently been able to isolate these cells from the peripheral blood [49,50]. In our experience, using an isolation and culture method similar to that used for BM-MSC, we would occasionally yield MSC-like cell colonies from the peripheral blood of adult mice or rats (unpublished data). Taken together, these studies suggest that circulating MSCs exist in the fetus and adults, but the abundance appears to decline markedly after birth. Notably, improvements in the isolation and culture methods have been shown to increase the yield of blood borne MSCs in culture [51]. Therefore, more efficient methods will enable determination of numerical and functional changes in circulating MSCs relating to age and disease in the future. It has been reported that hypoxia causes mobilization of BM-MSCs to the peripheral blood thus increasing the levels of circulating MSCs [52]. After wounding of the skin, putative BM-MSCs in the wound also increased [1,27]. Therefore, the number of circulating BM-MSCs was expected to increase accordingly, but unfortunately this has not been studied.

## BM-DERIVED MSCS DIFFERENTIATE INTO KERATINOCYTES

Recent studies have shown that cells derived from the BM not only contribute to fibroblast-shaped cells in the dermis but also cells in the epidermis such as a keratinocyte phenotype [1–3,53,54]. In one study, BM-derived epidermal cells were found to proliferate in the epidermis and localized to a known stem cell niche, that is, the CD34-positive bulge region of hair follicles in mice [3]. However, CD34-positive cells derived from the BM do not appear to contribute to CD34-postive stem cells in the bulge region of hair follicles [6]. Although theoretically BM-derived cells can form fusion cells with keratinocytes, and the event was indeed suggested to occur in one ex vivo study [55], several recent studies have shown that the event appears not to occur in vivo and cell differentiation accounts for the formation of BM-derived keratinocytes in vivo [6,54,56]. Using a Cre/lox transgenic mouse model together with β-galactosidase and EGFP expression, the classic system to determine cell fusion, BM-derived cells (including MSCs) were found to differentiate into epithelial cells in the skin, liver, and lungs without cell fusion [56]. The BM contains several cell types. To determine which cell type contributes to keratinocytes, we implanted GFP-expressing CD34-negative BM-MSCs or CD34-positive HSCs into wounds and examined the outcome. Our data showed that BM-MSCs but not HSCs adopted a keratinocyte phenotype, through cell differentiation [6].

## MSCS CONTRIBUTE TO STRUCTURAL REGENERATION OF THE SKIN

**Figure 1:**
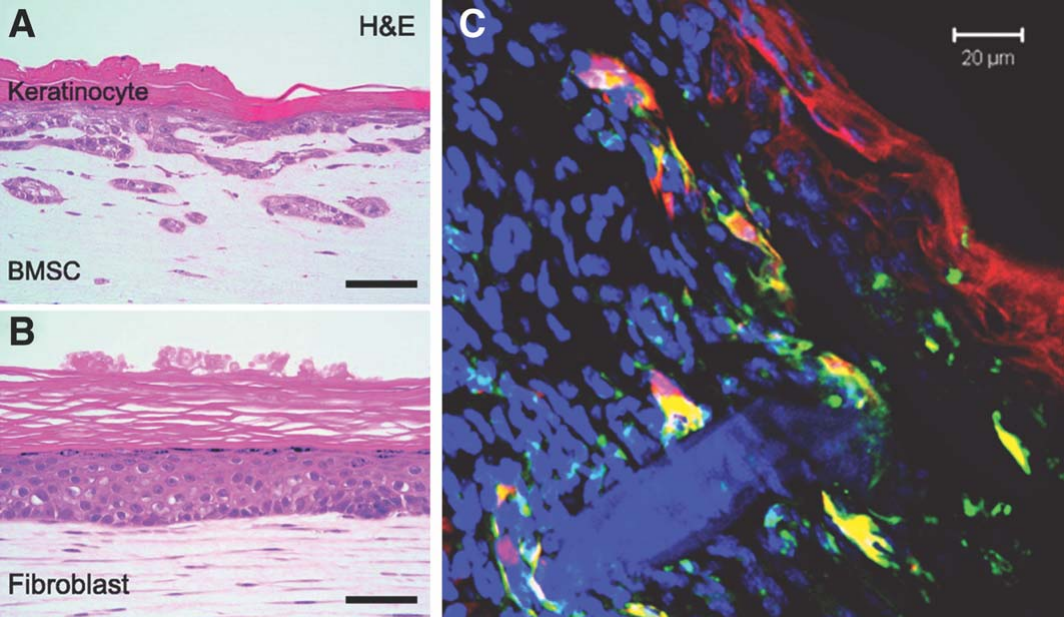
Bone marrow-derived mesenchymal stem cells (BM-MSCs) in cutaneous regeneration. **(A, B):** Keratinocytes loaded on a collagen gel containing BM-MSCs formed rete ridge-like structure, whereas keratinocytes loaded on a collagen gel containing dermal fibroblasts did not. Images adapted from Aoki et al. [58]. Image courtesy of Molecular Biology of the Cell. **(C):** BM-MSCs (green) from GFP-expressing mice were injected around the excisional wound and applied on the wound bed in Matrigel in Balb/C mice. At day 7, some mesenchymal stem cells (yellow) expressed keratinocyte marker cytokeratins (red) and formed structures similar to those observed in the study of Aoki et al. **(A)**. Abbreviations: BMSC, bone morrow-derived mesenchymal stem cells; H&E, hematoxylin and eosin stain.

In postpartum humans, injury to the skin and other tissues heals not by the regeneration of the tissue to the preinjured form but by the formation of scar tissue. Epidermal appendages that have been lost at the site of damage do not regenerate. A major goal of wound healing biology is to understand how skin can be induced to reconstruct the damaged portions without scar. Increasing evidence suggest that BM-MSCs contribute to cutaneous regeneration. In our previous study, following i.v. infusion of Flk1-positive BM-MSCs derived from Balb/C mice (with white hair) and unfractionated BM cells from female C57BL/6 mice for hematological reconstitution to lethally irradiated female C57BL mice (with black hair), the recipients gradually grew white hair. Immunohistochemical analysis of the skin areas bearing the white hair showed that follicles with white hair segments contained cells derived from donor MSCs [2], suggesting that BM-MSCs are significantly involved in the regeneration of functional hair follicles. Irradiation injuries to the skin result in cell death, but the framework of the cutaneous structure remains, which provides a scaffold for cutaneous regeneration. The involvement of BM-MSCs in cutaneous regeneration in excisional wounds, where the scaffold of the skin is lost along with the cells, has been addressed in the following studies. When a mixture of BM cells and embryonic skin cells were applied onto a fresh excisional wound bed within a wound chamber in nude mice, hair grew from the wounded areas, and a substantial amount of donor BM-derived cells were detected in the epidermis, hair follicles, and sebaceous glands. But when BM cells alone were transplanted onto the wound within the chamber, cutaneous regeneration did not occur [57]. However, when we transplanted BM-MSCs in a matrix gel onto excisional wounds without the chamber, glandular appendage-like structures largely formed by cells derived from BM-MSC appeared in the healing wound. These results suggest that BM-MSCs require the coexistence of skin cells and probably molecules released by them for differentiation and structural regeneration. This statement is further supported by ex vivo results. Coculture of irradiation injured keratinocytes with BM-MSCs significantly increased the expression of epithelial marker cytokeratins in BM-MSCs, compared with cocultures with uninjured keratinocytes [6]. Moreover, BM-MSCs and keratinocyte crosstalk appears also to be necessary for structural organization of the epidermis. In a previous study by Aoki et al., where keratinocytes were seeded on the top of a collagen matrix containing BM-MSCs, subcutaneous preadipocytes, or dermal fibroblasts, while all three types of stromal cells promoted the survival of keratinocytes, only BM-MSCs promoted the formation of rete ridge-like structure in the epidermis, which extended deeper into the matrix (resembling the dermis) forming glandular structures [58] (Fig. 1). Interestingly, similar glandular structures were also observed in our in vivo study in the healing wound, but some of the structures were formed by cells derived from the transplanted BM-MSCs, which resembled developing sweat or sebaceous glands; however, these structures disappeared when the wound was completely closed [6]. Consistent with our findings, epithelial cells derived from endogenous BM cells were found to appear in the wound transiently during wound healing [54]. These data suggest that BM may not provide long-term self-renewal stem cells for dermal keratinocytes. However, these temporary structures formed by keratinocytes derived from BM-MSCs may serve as precursors of endogenous permanent cutaneous structures and therefore play a critical role for cutaneous regeneration.

There are contradictory reports over the incidence of differentiation of BM-MSCs into epithelial cells [59,60]. As discussed in a recent review by Phinney and Prockop [61], a large number of variables are likely to contribute to the inconsistencies in these observations. With culture methods currently used by most research groups, BM-MSCs quickly age during culture expansion [55,62], and undergo autonomous differentiation toward osteoblasts [62]. Not surprisingly, a recent study showed that BM-MSCs released less vascular endothelial growth factor (VEGF) and exhibited decreasing protective effect to isolated rat hearts with successive passages [63]. The multipotent differentiation potential of BM-MSCs is expected to decrease accordingly. Therefore, to improve current culture conditions to better retain the primitive properties of MSCs has become a critical issue to achieve reproducible results in laboratory research and in clinical therapies in the future.

## CULTURE EXPANDED BM-MSCS ENHANCE WOUND HEALING

After wounding, a considerable number of BM-derived fibroblast-shaped cells are present in the skin, and as the major fibroblast-shaped cell population in the BM, BM-MSCs are likely to be the major component. Unlike HSCs, BM-MSCs can easily be expanded in culture. We and others have shown that ex vivo expanded BM-MSCs promote wound healing in several animal studies [29,64–67] (Table 2). Recent studies by ourselves and others indicate that the excisional wound splinting model can greatly restrain local skin contraction in rodents allowing the wound to heal through granulation and re-epithelialization, and thus give rise to uniform data [6,29,68,69]. With this model, we have shown that topically applied allogeneic BM-MSCs can significantly enhance wound healing in diabetic *db/db* mice and normal mice. BM-MSC-treated wounds exhibited significantly faster wound closure, with increased re-epithelialization, cellularity, and angiogenesis. Of note, allogeneic BM-MSCs were much more potent in promoting wound healing than allogeneic dermal fibroblasts, the major stromal cell population in the skin [6]. More recently, BM-MSCs have been shown to accelerate wound healing in diabetic rats [67]. Impressively, allogeneic BM-MSCs exhibited similar survival, engraftment, and effect as syngeneic BM-MSCs in promoting wound healing [65,70]. These data are of particular significance in developing MSC-based therapies, as recent studies have shown that biological activities and therapeutic potential of BM-MSCs are impaired in elderly individuals and patients with chronic diseases such as diabetes [71–75].

**Table 2:** Activities of bone marrow-derived mesenchymal stem cells in wound healing

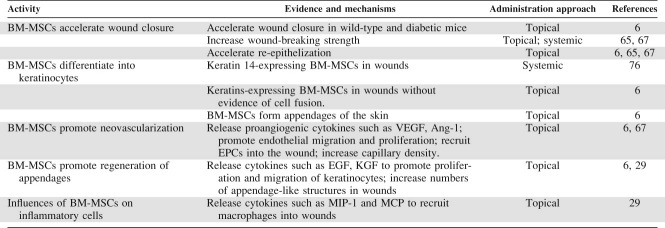

Abbreviations: BM-MSCs, bone marrow-derived mesenchymal stem cells; EGF, epidermal growth factor; EPC, endothelial progenitor cell; KGF, keratinocyte growth factor; MCP, monocyte chemoattractant protein; MIP-1, macrophage inflammatory protein-1; VEGF, vascular endothelial growth factor.

In addition to accelerating wound closer, BM-MSCs have been shown to improve the quality of cutaneous repair. Systemic administration of BM-MSCs significantly increased the wound bursting strength of fascial and cutaneous wounds [65]. More importantly, BM-MSCs appear to enhance cutaneous regeneration. In addition to differentiating into keratinocytes and forming appendage-like structures, BM-MSCs in the wound enhance the proliferation of endogenous keratinocytes and increase the number of regenerating appendage-like structures [6].

Little information is available about the effect of BM-MSCs in wound healing in humans. In a recent report, five patients with acute wounds and eight patients with chronic, long-standing, nonhealing lower extremity wounds received treatments with BM-MSCs. Autologous BM-MSCs were culture expanded and topically applied up to four times to the wounds in a matrix of fibrin. Subsequent tissue biopsy analysis showed signs of the survival of implanted BM-MSCs and generation of new elastic fibers in the wounds. A reduction of chronic wound size was found to be closely associated with the number of cells applied and no treatment-related adverse events were observed [7]. Although the results are encouraging, many questions remain, such as the optimal cell number per treatment, frequency of treatment, appropriate extracellular matrix (ECM) molecules for cell delivery, and the fate of the MSCs in the wound. Of these issues, ECM molecules used to deliver MSCs should be critical, as the microenvironment for MSCs to survive in human chronic wounds is very likely to be worse than that in animal models. Appropriate ECM molecules will not only promote the survival of MSCs in the wound but also provide materials required for wound healing.

## PARACRINE FACTORS OF MSCS IN CUTANEOUS REPAIR/REGENERATION

As stromal cells in the BM, MSCs have been known to support the survival, growth, and differentiation of HSCs by providing paracrine factors and ECM molecules. Therefore, MSCs residing in the skin or recruited into the wound are likely to play a role in maintaining the structural and functional integrity of the skin through a paracrine mechanism. Several studies have shown that BM-MSCs secrete a variety of cytokines [29,77,78]. In an antibody-based protein array analysis of 79 human cytokine including growth factors and chemokines, BM-MSC-conditioned medium reacted to the large majority of them [29]. Optimum healing of a wound requires a well-orchestrated integration of many molecular events mediated by cytokines. As fibroblasts are a major stromal cell population in the skin and are known to secrete diverse molecules involved in cutaneous homeostasis and wound healing [31,32], it is therefore of great significance to understand what distinctive roles the paracrine molecules of BM-MSCs play in the skin in contrast to dermal fibroblasts. As shown in a comparative analysis of BM-MSCs-conditioned medium versus dermal fibroblasts-conditioned medium, of 81 cytokines analyzed, 31 cytokines were distinctively expressed (Table 3). BM-MSCs secreted significantly larger amounts of several growth factors known to enhance normal wound healing [31,79,80], but significantly lower levels of interleukin-6 (IL-6) and osteoprotegerin than dermal fibroblasts. Of the differentially expressed growth factors, insulin-like growth factor-1 (IGF-1) is particularly intriguing as the expression of IGF-1 in BM-MSCs is extremely high and IGF-1 has recently been shown to play a critical role in the regeneration of various tissues [81,82]. Therefore, IGF-1, along with other differentially expressed cytokines, may be involved in cutaneous regeneration. In accordance with this speculation, BM-MSC-conditioned medium significantly promoted the proliferation of keratinocyte and endothelial cells in vitro [6], and BM-MSC-treated wounds contained larger numbers of skin appendages compared with dermal fibroblast-treated wounds [6]. IL-6 has long been known as a potent proinflammatory cytokine [83]. Osteoprotegerin is involved in bone metabolism [84] and its role in wound healing remains to be defined. In general, cytokine expression profiling suggests that BM-MSCs secrete higher levels of cytokines known to enhance cell growth and tissue regeneration, whereas dermal fibroblasts expressed greater amounts of cytokines known to promote inflammation. It is likely that there are higher levels of circulating and residential MSCs in the human newborn, which mediate more regeneration after wounding.

**Table 3:** Differentially expressed paracrine factors in bone marrow-derived mesenchymal stem cells versus dermal fibroblasts

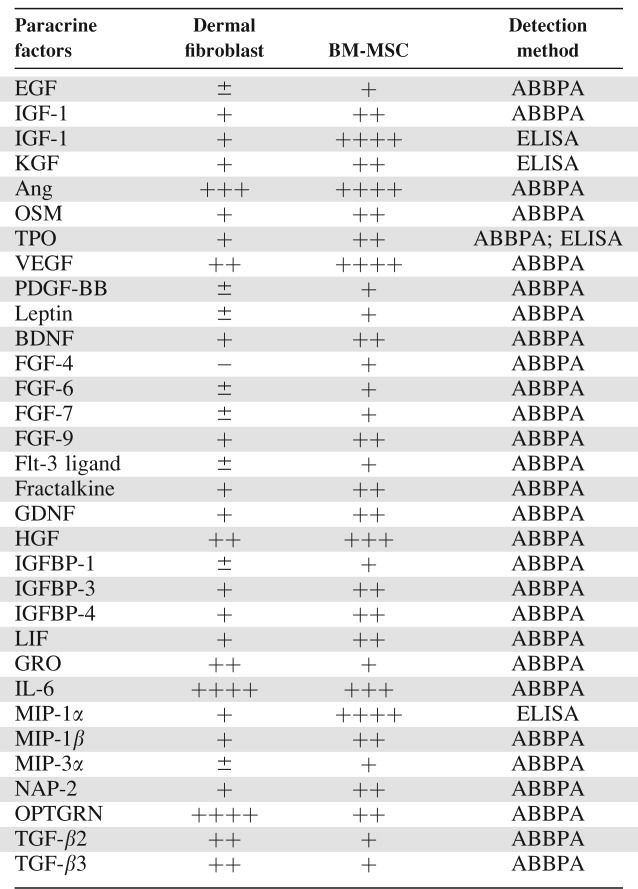

Human dermal fibroblast- or BM-MSC-conditioned medium under hypoxic conditions for 24 hours was analyzed with ABBPA and ELISA. The intensity of each dot was measured. “−”, not detected; ±, weakly detected; + to ++++, intensity of positive detection.

Abbreviations: ABBPA, antibody-based protein array; Ang; angiopoietin; BDNF, brain-derived neurotrophic factor; BM-MSC, bone marrow-derived mesenchymal stem cell; EGF, epidermal growth factor; ELISA, enzyme-linked immunosorbent assay; FGF, fibroblast growth factor; Flt-3 ligand, FMS-related tyrosine kinase 3 ligand; IL, interleukin; GDNF, glial cell line-derived neurotrophic factor; GRO, growth-related oncogene; HGF, hepatocyte growth factor; IGF, insulin-like growth factor; IGFBP, IGF binding protein; KGF, keratinocyte growth factor; LIF, leukemia inhibitory factor; MIP, macrophage inflammatory protein; NAP-2, neutrophil-activating peptide; OPTGRN, osteoprotegrin; OSM, oncostatin M; PDGF-BB, platelet-derived growth factor-BB; TGF, transforming growth factor; TPO, thrombopoietin; VEGF, vascular endothelial growth factor.

**Figure 2:**
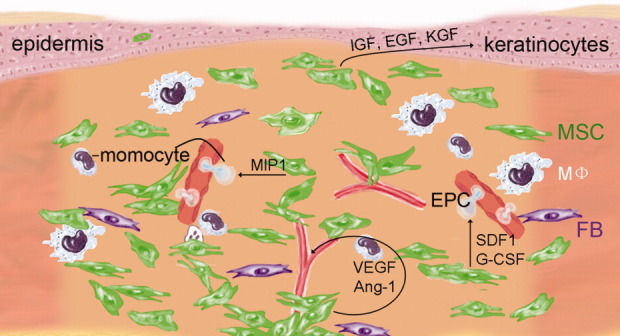
Paracrine effect of Bone marrow-derived mesenchymal stem cells (BM-MSCs) in wound healing. BM-MSCs (green) in a cutaneous wound release growth factors such as IGF-1, EGF, and KGF to promote the proliferation of keratinocytes, release proangiogenic cytokines such as VEGF-a and Ang-1 to enhance angiogenesis, release chemokines such as MIP-1 to recruit monocytes into the wound, and release cytokines such as SDF-1 and G-CSF to recruit EPCs into the wound. Abbreviations: ang-1, angiopoietin-1; EGF, epidermal growth factor; EPC, endothelial progenitor cell; FB, fibroblast; G-CSF, granulocyte colony-stimulating factor; IGF, insulin-like growth factor; KGF, keratinocyte growth factor; MIP-1, macrophage inflammatory protein-1; MSC, mesenchymal stem cell; SDF1, Stromal cell-derived factor-1; VEGF, vascular endothelial growth factor.

Inflammatory cells actively participate in wound healing. Immediately after injury, the wound is hypoxic, which creates a chemoattractive environment to circulating inflammatory cells. Specific types of leukocytes are recruited into the wound after acute injuries, with neutrophils, monocytes (macrophages), and lymphocytes sequentially dominant in the wound [31,85], and disturbances in this orderly procedure may cause disorders in wound healing. For example, delayed neutrophil and monocyte infiltration but more sustained lymphocyte infiltration has been considered an important causative factor of chronic wounds [85,86]. In addition to fighting bacteria and cleaning up tissue debris from the wound, inflammatory cells clearly affect the wound healing process through paracrine cytokines [31,85]. But recent knockout and knockdown studies in mice suggest that certain leukocyte lineages are not essential for the healing of cutaneous wounds, particularly a wound without infection. For instances, loss of mast cells in the wound speeds wound healing with reduced scaring and loss of neutrophils appears not to affect wound healing [87]. BM-MSCs are capable of releasing a large number of chemokines such as macrophage inflammatory protein (MIP)-1, MIP-1, MIP-2, monocyte chemoattractant protein (MCP)-5, stromal cell-derived factor (SDF)-1, and G-CSF, which are known to affect certain inflammatory cell lineages. It is therefore of importance to understand whether transplantation, particularly local application, of BM-MSCs influences the inflammation process in wound healing. Reduced infiltration of inflammatory was found in the infarcted myocardium receiving local injection of BM-MSCs, which was considered to contribute to the reduced scarring of the myocardium, but which population of inflammatory cells was affected was not examined [88]. Analysis of cells per excisional wound indicated that BM-MSC-conditioned medium significantly increased the number of macrophages but unchanged the amount of granulocytes were found; T-cell levels showed a trend of modest reduction [6]. The results are consistent with in vitro data, in which BM-MSC-conditioned medium was strongly chemoattractive to monocytes (sixfold greater than dermal fibroblast-conditioned medium) [29]. MIP and MCP are major chemoattractants for monocytes/macrophages and play a key role in macrophage infiltration during wound healing [89,90]. BM-MSCs release several fold greater amounts of MIP-1 and MCP-5 than dermal fibroblasts [29]. Tissue macrophages have been known to play a pivotal role in wound healing [89–92]. These results suggest that BM-MSCs selectively recruit monocytes into the wound through a release of chemoattractive cytokines (Fig. 2), implying a significant therapeutic value of BM-MSCs in chronic wounds.

Neovascularization is a crucial step in the wound healing process [31,79,93]. Local application of BM-MSCs promotes neovascularization [6]. Paracrine factors of BM-MSCs appear to play a major role for the increased angiogenesis. First, BM-MSCs are capable of releasing high levels of proangiogenic cytokines such as VEGF-α, IGF-1, platelet-derived growth factor-BB, and angiopoietin (Ang-1), and increased amounts of VEGF-α and Ang-1 were detected in wounds treated with BM-MSCs [29,66]. Second, BM-MSC-condition medium enhanced angiogenesis in vitro [6]. Finally, wounds treated with BM-MSC-conditioned medium had increased numbers of cells positive for CD34, C-kit, or Flk-1, markers for endothelial lineage cells, suggesting increased recruitment of endothelial cells and EPCs into the wound [29] (Fig. 2).

## BM-DERIVED EPCS IN WOUND HEALING

EPCs are generally considered to be lineage cells derived from HSCs in the BM, which express CD34 and certain endothelial lineage markers such as Flk-1 and CD31 on the surface [94]. Currently, it is understood that circulating EPCs contribute to a proportion of endothelial cells in adults [10,95–97]. Recently, it has been found that the release of HSCs from the BM is under circadian control, with a clear peak of HSC count in the peripheral blood [98,99]. A similar circadian rhythm has been observed with EPCs, and disturbances in this rhythm in diabetes may be associated with pathogenesis of retinopathy [100]. These observations have implications with respect to wound healing. It may be advantageous to schedule surgeries in at risk patients for the time of day when circulating EPCs to promote healing are at their maximal levels. Reduced numbers of circulating EPCs have been found in a broad spectrum of cardiovascular diseases including coronary artery diseases [101,102] and rheumatoid arthritis [103]. In addition to numerical changes, impaired function of circulating EPCs has been found to be associated with cardiovascular diseases and aging [104]. EPCs can be mobilized into the peripheral blood by cytokines such as stromal cell derived factor-1 (SDF-1), VEGF, and G-CSF [10,105,106], vascular trauma [107], and ischemia [10]. After systemic infusion, EPCs can home to ischemic tissue, where they enhance neovascularization and improve functional performance of the organ [10,96,97].

Recent studies suggest EPCs play an important role in wound healing. Transplantation of EPCs enhances wound healing in mice [108]. Wounds treated with EPCs exhibited accelerated wound closure and increased vascular density. Despite evidence indicating that EPCs contribute to a certain portion of normal endothelial cells, recent studies have shown that direct contribution of endothelial cells may not be a major mechanism in EPC-mediated enhanced wound healing. Instead, paracrine factors of EPCs appear to play a central role [108]. EPCs have been known to release a variety of growth factors such as VEGF, hepatocyte growth factor, G-CSF, GM-CSF [109], and PDGF [110]. In a more recent study, topical application of EPCs to ischemic wounds of diabetic mice resulted in accelerated wound closure with increased angiogenesis in the wound, and the action was abrogated by coadministering the Wnt antagonist secreted frizzled-related protein-1 or neutralizing antibodies against VEGF-α or IL-8, implying that EPCs stimulate wound healing by paracrine mechanisms that activate Wnt signaling pathway in recipients [111]. Consistent with the therapeutic effect of EPCs on wound healing in animals, reduced numbers and impaired function of circulating EPCs have been described in both type 1 and type 2 diabetic patients [112,113]. EPCs derived from diabetic mice exhibited impaired vascularization and wound healing [114]. Moreover, chronic wounds in patients with diabetes showed reduced chemoattractive ability to EPCs. Administration of exogenous SDF-1α into the wound reversed EPC homing into the wound and promoted wound closure [115].

## FIBROCYTES IN WOUND HEALING AND FIBROSIS

Fibrocytes originate from the BM and are considered to be a newly identified leukocyte subpopulation. They constitute 0.1%–0.5% of peripheral blood cells and exhibit both monocyte and fibroblast-like characteristics [116,117] and are characterized by the expression of collagen type I, fibronectin, CD11b, CD34, and CD45 but not CD14, CD3, or CD10. Fibrocytes were initially discovered by their rapid and specific recruitment from blood to implanted wound chambers in mice [118]. Subsequently, they have been found in the peripheral blood, wound sites, and areas of tissue remodeling [118].

Numerous fibrotic diseases have been found to be associated with the presence of fibrocytes in fibrotic tissues including renal fibrogenesis [119], liver fibrosis, and bleomycin-mediated pulmonary fibrosis. They have been reported to contribute to the myofibroblast population in wounds [120]. The number of fibrocytes has been found to be significantly increased in burn patients (up to 10% of peripheral blood mononuclear cells) compared with that of normal individuals (<0.5%). Moreover, increased amounts of fibrocytes were found in hypertrophic scar tissue located primarily in the deeper layers of the papillary dermis [121], and were found to produce more collagen but less collagenase, compared with fibroblasts in the top layer of the dermis, suggesting a role of fibroblasts from the deeper layer of dermis in the development of fibrosis [122]. It is likely that fibrocytes regulate the function of fibroblasts in the deeper dermis in a paracrine fashion through cytokine production. It has been shown that fibrocytes are potent producers of profibrotic cytokines transforming growth factor-1 and connective tissue growth factor. In addition, fibrocytes can produce ECM molecules such as type I and III collagen and fibronectin by themselves [117,118,123]. Consistent with these observations, it has been shown that circulating fibrocytes homed to bleomycin-induced lung inflammation, differentiated into fibroblasts and contributed to lung fibrosis [8]. Interesting but not surprisingly, systemic administration of BM-MSCs inhibits bleomycin-induced lung inflammation and collagen deposition [124]. Taken together, fibrocytes originating from the BM interact with surrounding cells in the skin and may play a pivotal role in abnormal healing such as hypertrophic scarring.

## CONCLUSIONS

Recent studies suggest that “fibroblasts” in the dermis are heterogenous in origin and function, and the conventional understanding of these cells, which is based largely on cell morphology is inadequate. In addition to fibroblasts that remain during embryonic development, BM appears to be an important source that provides “fibroblasts” to the skin in adults, which may include, but is not limited to, CD45-negative MSCs, CD45-positive fibrocytes and EPCs. The role of these newly discovered components of “fibroblasts” in the dermis are not fully understood. BM-MSCs and EPCs are thought to enhance cutaneous repair/regeneration and fibrocytes appear to cause fibrosis and are probably involved in hypertrophic scar formation. In the future, the development of specific markers to recognize MSCs and fibrocytes in vivo and animal models deficient for these cells will allow us to better understand their role in physiology and in diseases.
